# The Impact of Bilingualism on Executive Functions in Children and Adolescents: A Systematic Review Based on the PRISMA Method

**DOI:** 10.3389/fpsyg.2020.574789

**Published:** 2020-10-06

**Authors:** Jasmine Giovannoli, Diana Martella, Francesca Federico, Sabine Pirchio, Maria Casagrande

**Affiliations:** ^1^Dipartimento di Psicologia, Sapienza Università di Roma, Roma, Italy; ^2^Instituto de Estudios Sociales y Humanísticos, Universidad Autónoma de Chile, Santiago de Chile, Chile; ^3^Dipartimento di Psicologia e dei Processi di Sviluppo e Socializzazione, Sapienza Università di Roma, Roma, Italy; ^4^Dipartimento di Psicologia Dinamica e Clinica, Sapienza Università di Roma, Roma, Italy

**Keywords:** bilingualism, executive functions, bilingual advantage, inhibition, shifting, working memory

## Abstract

Approximately half of the world's population is bilingual or multilingual. The bilingual advantage theory claims that the constant need to control both known languages, that are always active in the brain, to use the one suitable for each specific context improves cognitive functions and specifically executive functions. However, some authors do not agree on the bilingual effect, given the controversial results of studies on this topic. This systematic review aims to summarize the results of studies on the relationship between bilingualism and executive functions. The review was conducted according to PRISMA-statement through searches in the scientific database PsychINFO, PsycARTICLES, MEDLINE, and PUBMED. Studies included in this review had at least one bilingual and monolingual group, participants aged between 5 and 17 years, and at least one executive function measure. Studies on second language learners, multilingual people, and the clinical population were excluded. Fifty-three studies were included in the systematic review. Evidence supporting the bilingual effect seems to appear when assessing inhibition and cognitive flexibility, but to disappear when working memory is considered. The inconsistent results of the studies do not allow drawing definite conclusions on the bilingual effect. Further studies are needed; they should consider the role of some modulators (e.g., language history and context, methodological differences) on the observed results.

## Introduction

Approximately half of the world population is bilingual or multilingual (Ansaldo et al., 2008). In 2016, 64.6% of the European population aged 25–64 declared they knew one or more foreign languages. When considering only 25–34-year-olds, this percentage rises to 73.3% (Eurostat, 2016). Moreover, the number of immigrant children worldwide who do not speak the majority language of their place of residence has increased (OECD, 2010). Despite that, there is no single definition of bilingualism. Among the definitions of bilinguals, the most inclusive is the one by Edwards (2004), who states that “everyone is bilingual” because there are no (adult) people in the world who do not know at least some words in a language different from their native language. According to other definitions (Abdelgafar and Moawad, 2015), only people who know two languages with a level of competence equal to that of a native speaker can be considered bilingual. The more common definition is “someone who can function in both languages in conversational interaction” (Wei, 2020). The age of acquisition (AoA) of the second language is another factor that characterizes bilinguals, allowing to classify them in simultaneous bilinguals, when both languages are learned during infancy, and sequential bilinguals, when they are exposed to the second language after infancy, usually at school entry (Gross et al., 2014). Other authors also include learning a second language as they define bilinguals who can correctly produce sentences in a language other than their native language (Hakuta, 1986). The absence of standard guidelines has led to heterogeneity in the populations considered by studies on bilingualism, often including people with different language histories and competencies (for a list of terms used to describe bilinguals, see Wei, 2020).

The first studies on bilingualism date back to the early 1900s. Initially, several researchers supported the hypothesis that bilingual children had lower mental abilities than monolinguals because the knowledge of several languages would generate a mental confusion with deleterious consequences on every cognitive aspect (Hakuta, 1986). Peal and Lambert (1962) were the first to contradict this negative view about the bilingualism effect. Because of the positive results of subsequent studies, a new theory advanced the view of bilingualism advantage. The positive effect of bilingualism would depend on the constant need to control both known languages to use the one suitable for each specific context, and this process would generate more significant neurological development (Bialystok, 1999, 2001). According to the *Joint Activation Model* of Green (1998), both languages would always be active in the brain of a bilingual person regardless of the language used at the given moment; for this reason, it would be necessary to use a general suppression mechanism to inhibit the activation of the non-target language. Green and Abutalebi (2013) highlighted the importance of the context in which language exchanges take place. They proposed the *Adaptive control hypothesis* and identified three possible contexts of interaction: single-language, dual-language, and dense code-switching contexts. Depending on the communicative context in which bilinguals are immersed, the languages may cooperate or compete. For this reason, each context is characterized by a different use of processes that are the basis of communication. The use of multiple languages would seem to modify both the language network and the control network (Green and Kroll, 2019).

Some of the cognitive functions that would seem to benefit from the knowledge of several languages are the metalinguistic and metacognitive awareness, the ability to represent abstract and symbolic concepts (for a review see Adesope et al., 2010), and specifically, the bilingualism should improve the executive functioning.

According to the model of Miyake et al. (2000), executive functions refer to cognitive flexibility (e.g., the ability to switch between tasks), inhibition (e.g., the ability to suppress dominant responses) and monitoring (e.g., the ability to update information in the working memory).

According to Bialystok (2011), bilinguals have an advantage in executive functions because they would continuously train them to carry on a conversation that must be based on the context and require constant access to the information contained in the working memory. Furthermore, it is necessary to select the appropriate language for the specific communicative situation (inhibiting the other language) and to monitor what happens during the interaction (cognitive flexibility).

It has been shown that executive functions can be improved through training (Karbach and Kray, 2009; Moreno et al., 2011). The study of the “bilingual advantage” is not only one of the main topics discussed in bilingualism research, but it is also the most controversial one. After the publication of positive evidence on the bilingual advantage, the difficulty in replicating previous results and the publication of several studies with null findings led to questioning this theory. Recently, the use of the term “bilingual advantage” has been questioned because its presence or absence could depend on the interpretation or perspective of the observer. Leivada et al. (2020) suggested adopting the more neutral term “bilingual effect.” Paap et al. (2015) stated that “bilingual advantages in executive functioning either do not exist or are restricted to very specific and undetermined circumstances” and pointed out that 80% of the tests carried out after 2011 failed to obtain results in support of the bilingual effect.

Paap et al. (2015) hypothesized that the results of previous studies on this topic could be due to the lack of control of several external factors, the experimental tasks chosen to evaluate it, and the limited number of participants included in the studies. Other factors that play a role in determining these results are socioeconomic status (SES) and the participants' cultural and linguistic background. For example, the tests used for the assessment of bilinguals are usually the same as those used and validated for monolinguals. The condition of bilingualism can influence the performance in various domains (positively or negatively). In that case, it follows that some of the standardized tests currently in use are not always suitable for the assessment of bilinguals and that the normative data currently available do not reflect the real abilities of bilinguals (e.g., assessment of linguistic abilities in bilingually developing children, see for example Core et al., 2013; Bailey et al., 2020). One of the characteristics of the experimental tasks that seem to influence the performance of people who know several languages is the use of verbal stimuli (Duñabeitia et al., 2014).

Many studies have shown that bilinguals perform more poorly than monolinguals on linguistic tasks (e.g., Bialystok, 2009a), have a smaller vocabulary than monolinguals (Bialystok et al., 2010) and produce fewer words in verbal fluency tasks (Zeng et al., 2019). These findings could be due to the lower use and the specificity of each language. The characteristics of the two languages could depend on how they were learned and used (Blom et al., 2014). When the vocabulary size is assessed considering both known languages, this deficit disappears, and bilinguals show a more extensive vocabulary size than monolinguals (Bialystok, 2009b).

The use of verbal stimuli implies the activation in the brain of bilinguals of two different linguistic forms per stimulus and difficulty in coding when the presented word is known in the other language than the one used for the assessment. Other factors related to language skills seem to affect the performance of bilinguals. In tasks using verbal stimuli, both the similarity of the languages known and the native language would seem to affect the results. Unfortunately, however, for many of the aspects of the linguistic experience, there is still no agreed conclusion between the different researchers. For instance, what is the degree of balance that must exist between the two languages to generate the bilingual effect? Some studies argue that the bilingual effect emerges when bilinguals have complete mastery of the two languages (Filippi et al., 2015). Therefore, the advantage should be due to the higher cognitive effort needed to reduce interference between the two languages (Blom et al., 2014); other researches asserted that the potential cognitive effects are proportionate to the degree of balance between languages (Carlson and Meltzoff, 2008; Ladas et al., 2015).

Other authors argue that the degree of control that bilinguals must apply is higher when they are not equally fluent in the two languages; therefore, the absence of significant differences in the studies could be due to the inclusion of participants with a balanced competence in the two languages for whom the process of switching has become automatic (Gathercole et al., 2014). A factor that does not seem to affect the degree of advantage in executive functioning is the knowledge of more than two languages (Poarch and van Hell, 2012; Poarch and Bialystok, 2015). The type of language known and the degree of similarity between them is also an aspect to be considered. Several authors have pointed out that the similarity between languages is a decisive factor in determining the bilingual effect (e.g., Bialystok et al., 2003), while phonological and orthographic differences can negatively affect performance, generating interference during the evaluation (Jalali-Moghadam and Kormi-Nouri, 2015).

There are also specific characteristics of the experimental tasks that seem to affect the performance of bilinguals. Several studies agree that the bilingual effect would emerge in more complex experimental tasks where there is a higher demand for control (e.g., Engel de Abreu et al., 2012; Barac et al., 2016). Further, the tendency to use experimental tasks that empirically isolate executive functions seems to contribute to unclear results (Barac et al., 2016). Most experimental tasks inevitably engage other cognitive processes while evaluating a specific domain (task impurity problem; Miyake and Friedman, 2012). Isolating the executive functions experimentally also does not allow the evaluation of real conditions since, in daily life, rarely exist tasks involving a single component of cognitive functions. Another aspect to consider is test-retest reliability. Several experimental tasks used to evaluate executive functions are characterized by low test-retest reliability, and this factor should lead to a more cautious interpretation (Karalunas et al., 2016; Leivada et al., 2020). Additionally, bilingualism seems to have a more significant impact when it is required to coordinate multiple functions simultaneously (Bialystok, 2011).

Other factors, such as socioeconomic status, cultural aspects, or immigrant status, would seem to have a role in determining the results achieved by bilingual participants. In several American countries, the condition of bilingualism is a consequence of migratory phenomena, and it is associated with low socioeconomic status (Calvo and Bialystok, 2014). In other countries, for example, in Arab Countries, bilinguals usually belong to a high social class and often learn more than one language because they receive a bilingual school education (Abdelgafar and Moawad, 2015). It is known that low socioeconomic status leads to lower cognitive functioning (Rosen et al., 2019). Given the high frequency of low socioeconomic status and reduced vocabulary in bilinguals, several authors have indicated the importance of analyzing these aspects and monitoring the effect of these variables statistically if a difference between groups is present. Although many authors considered that statistical control of these variables is the correct procedure (e.g., Carlson and Meltzoff, 2008; Blom et al., 2014), others believe that these conditions are a specific characteristic of the population of interest (Buac et al., 2016).

This systematic review aims to summarize the findings of studies investigating the relationship between bilingualism and executive functions in children and adolescents. It will be verified whether bilingualism affects one or more specific executive functions. Studies that have used the same task will be compared, highlighting any changes that have been made to the experimental tasks that could influence the results. The studies will be analyzed to identify any factors that may be involved in determining the outcomes. We excluded studies with older adult participants from this systematic review, although they provide the strongest evidence for a bilingual effect (Antón et al., 2014). As Baum and Titone (2014) suggested, older adults experienced a historical and cultural moment in which attitudes toward bilingualism were very different from those of today. This factor could have affected the use of languages at various times in their lives. Moreover, studies with adults would imply the need to consider many other factors (e.g., drug treatment). We believe it is necessary to conduct a systematic review focusing only on this population, considering its specific characteristics.

## Method

The review process was conducted according to the PRISMA Statement (Liberati et al., 2009; Moher et al., 2009). The PRISMA Statement consists of a 27-item checklist and a four-phase flow diagram and helps authors improve systematic review reporting. This review was registered as PROSPERO CRD42019127965.

### Research Strategies

A systematic search of the international literature was conducted in the following electronic databases by selecting articles published in peer-review journals: PsycINFO, PsycARTICLES, MEDLINE, and PubMed. The last research was conducted on 15 April 2020. Restrictions were made limiting the research to academic publications in English, Italian, and Spanish. No restriction of age, gender, or ethnicity was made. The search strategy used Boolean combinations of the following keywords: “bilingual^*^,” “second language user,” “executive function^*^,” “cognitive flexibility,” “shifting,” “task switching,” “updating,” “working memory,” “inhibition,” and “cognitive inhibition.” Reference lists of the selected articles were screened. A total of 3,785 articles were obtained from the search procedure. Mendeley reference manager software was used for removing duplicates. The first screening was made by reading the title and abstract. The full text of the selected studies was read.

### Eligibility Criteria

The studies that respected the following characteristics were included: the presence of at least one bilingual group and one monolingual group, at least one executive function measured, age of participants between 5 and 17 years. Studies on preschool-age children were excluded because the EFs and underlying neural areas are immature and still developing (Diamond, 2013). The age limit has been set at 17 years because, during middle adolescence, the peak of executive functions is reached (Anderson, 2002). Studies on bimodal bilingual, second language learners, and trilingual or multilingual people were excluded. Studies on clinical populations were excluded. All the selected studies were screened to assess the risk of bias using Standard quality assessment criteria for evaluating primary research papers from various fields (Kmet et al., 2011). The studies were included if they reached a score above 70%.

### Data Collection

According to the PICOS approach (Liberati et al., 2009), the following information has been extracted from the selected studies: author(s) and year of publication, country, characteristics of participants (age, percentage of females, spoken languages, use of languages, socioeconomic status), criteria used for selecting bilingual participants, the experimental paradigm used, results of the studies. These data are summarized in Tables 1, 2.

**Table 1:** Main characteristics of the studies included.

**Authors**	**Participants**					**Country**	**Definition of bilingual group**	**Tasks**	**Results**
	**Group**	**N**	**Age mean (SD)**	**Sex (% female)**	**Language**				
Abdelgafar and Moawad (2015)	M B	25 25	8.9 (0.9) 8.6 (0.7)	52 56	Arabic Arabic–English	Saudi Arabia	Background language questionnaire -parents/caregivers.	Stroop task—Color naming Bondi et al. (2002) Verbal Fluency Task (Arabic) TMT-A TMT-B	Stroop, Verbal Fluency B equal to M TMT-A (time) B faster than M TMT-B (time) B equal to M
Antón et al. (2014)	M Group 1 Group 2 Group 3 B Group 1 Group 2 Group 3	180 60 60 60 180 60 60 60	n.r. 7.55 (0.53) 9.50 (0.60) 11.47 (0.54) n.r. 7.57 (0.59) 9.53 (0.57) 11.43 (0.65)	59 n.r. n.r. n.r. 58 n.r. n.r. n.r.	Spanish Spanish–Basque	Spain, Basque country	Linguistic competence questionnaire -parents	ANT (Rueda et al., 2004)	ANT RTs, ACC, Alerting, Orienting, Conflict B equal to M
Arizmendi et al. (2018)	M B	167 80	7.7 (0.4) 7.9 (0.5)	- -	English English–Spanish	Arizona	Parents/Caregiver had to report that: - child could carry on a conversation in English and Spanish;- English or Spanish was their primary language.- At least one primary caregiver spoke Spanish at home. - Academic instruction could have included English, Spanish or both.	Classic Stroop Task Cross-modal Stroop task Stop-Signal task Pirate Sorting task Global-Local task Number Updating task N-back Auditory task N-back Visual Task	Stroop Task, Stop-Signal Task, Pirate Sorting Task (RTs), Global-Local Task, Number Updating B equal to M N-Back Auditory and Visual B lower than M
Barac and Bialystok (2012)	M BCE BFE BSE	26 30 28 20	5.96 (0.52) 5.96 (0.54) 6.23 (0.32) 6.20 (0.82)	50 47 43 50	English Chinese–English French–English Spanish–English	n.r.	LSBQ (parents)	Color-shape task switching	Color-shape task switching (RTs) B faster than M Global Cost B lower than M
Barac et al. (2016)	M B	37 25	5.24 (0.47) 5.44 (0.43)	65 40	English English–Mixed	Canada	LSBQ (parents) Receptive-only bilinguals were excluded	Go/No-Go^a^ ANT (Rueda et al., 2004) Gift Delay with Cover	Go/No-Go Accuracy, D' B higher than M Go Trials (RTs) B faster than M ANT ACC incongruent, congruent trials B higher than M RTs, Alerting, Orienting, Conflict B equal to M Gift Delay with Cover B equal to M
Bialystok (1999)	M B	15 15	5.5 (n.r.) 5.5 (n.r.)	n.r. n.r.	English Cantonese/Mandarin–English	n.r.	Parents/Caregivers confirmed their status.	VCR (Zelazo et al., 1997) DCCS (Zelazo et al., 1996)	VCR B equal to M DCCS Postswitch phase, knowledge action phase B higher than M
Bialystok (2010)	Study 1 M B Study2 M B Study 3 M B	25 26 25 25 25 25	6.1 (n.r.) 6.0 (n.r.) 5.8 (n.r.) 5.8 (n.r.) 6.0 (n.r.) 6.1 (n.r.)	44 35 52 48 60 40	English English–Mixed English English–Mixed English English–Mixed	n.r.	LSBQ (parents)	Study 1 Category fluency Forward digit span TMT-A TMT-B Global-Local task Study 2 Same task as Study 1. Modified version of Global-Local task (control condition, fewer trials)	Study 1 Category fluency, digit span B equal than M TMT-A, TMT-B (time) B faster than M Global-Local task Accuracy Local condition B equal to M Global condition B higher than M RTs Global and local condition B faster than M
								Study 3 Same as study 1. No category fluency. Modified version of Global-Local task (block presentation of congruent trials). Backward Digit Span	Study 2 Category fluency, digit span B equal to M TMT-A, TMT-B (time) B faster than M Global-Local task all condition RTs B faster than M Study 3 Forward digit span M higher than B Backward digit span M equal to B TMT-A, TMT-B (time) B faster than M Block of congruent trial B equal to M Mixing cost B lower than M
Bialystok (2011)	M B	32 31	8.63 (0.31) 8.62 (0.28)	66 48	English English–Mixed	n.r.	LSBQ (parents)	Dual-modality classification task	Dual-modality classification task RTs B equal to M ACC (dual modality condition) B higher than M
Bialystok and Feng (2009)	M B	20 20	7.21 (0.65) 6.90 (0.33)	45 55	English English–Mixed	n.r.	Parent Questionnaire	Forward digit span Sequencing span Proactive Interference	Forward and sequencing digit B equal to M PI B equal to M PI Intrusions B lower than M
Bialystok and Viswanathan (2009)	M BC BI	30 30 30	8.5 (0.5) 8.5 (0.5) 8.6 (0.5)	50 50 60	English English–Mixed English–Tamil/Telugu	Canada Canada India	Parent Questionnaire	Animal span task Sequencing span task Corsi block TMT-A TMT-B Face task (Bialystok et al., 2006)	Animal/Sequencing span, Corsi BC, BI equal to M TMT-A (time) BI faster than BC BC equal to M TMT-B (time) BI equal to BC BC faster than M Face task Accuracy BI, BC equal to M RTs green, red eyes mixed presentation BI, BC faster than M Inhibitory control cost, Switching cost BI and BC lower cost than M Response suppression BI, BC equal to M
Blom et al. (2017)	M BFD BLD BPD	44 44 44 44	6.83 (0.58) 6.83 (0.50) 7.00 (0.50) 6.83 (0.58)	45 45 45 50	Dutch Frisian–Dutch Limburgish–Dutch Polish–Dutch	the Netherlands	PaBiQ (Tuller, 2015)	Backward digit span Dot Matrix task Sky Search task (Manly et al., 1999) Flanker Task (Engel de Abreu et al., 2012)	Digit span, Dot Matrix task, Flanker Effect B equal to M Sky search task B faster than M
Blom et al. (2014)	M Wave 2 Wave 3 B Wave 2 Wave 3	52 68	5.14 (0.17) 5.87 (0.20) 5.22 (0.22) 5.98 (0.24)	41 35	Dutch Turkish–Dutch	the Netherlands	Interview to mothers definition of bilingualism by Kohnert (2010)	Forward and Backward Digit Span Dot Matrix Task Odd-One-Out	5 anni (ANCOVA: SES, vocabulary) All tasks B equal to M 6 anni (ANCOVA: SES, vocabulary) Dot Matrix task, Backward Digit recall B better than M Odd-One-Out, Forward Digit Span B equal M
Bonifacci et al. (2011)	All M B	36 18 18	n.r. 9.61 (2.06) 9.28 (2.30)	44 n.r. n.r.	Italian Italian–Mixed	Italy	Italian parent + parent with a different L1 OR spoke an L1 different from Italian and had attended an Italian school for at least 6 years	modified Go/No-Go Memory with numbers Memory with symbols	Go/No-Go (Accuracy, RTs) B equal to M Memory with numbers/symbols B equal to M
Bosman and Janssen (2017)	M B	48 38	7.2 (0.6) 7.4 (0.6)	48 37	Dutch Turkish–Dutch	the Netherlands	Turkish at home, Dutch at school	Forward, Backward, Listening Recall	Forward, Backward, Listening Recall B lower than M
Buac et al. (2016)	M B	36 46	6.34 (0.84) 6.24 (0.76)	42 63	English Spanish–English	Madison, Wisconsin	Primary caregiver interview	Forward, Backward, Listening Recall Word Span Non word repletion	Forward Recall, Word Span, Non word repetition B lower than M Backward, Listening Recall B equal to M
Calvo and Bialystok (2014)	WCM MCM WCB MCB	20 46 44 65	6.67 (0.38) 6.70 (0.33) 6.80 (0.32) 6.66 (0.35)	55 46 57 49	English English English–Mixed English–Mixed	Toronto, Canada	LSBQ (parents)	Pair Cancelation (Woodcock et al., 2001) Cancelation (WISC-IV; Wechsler, 2003) Flanker Task Frog Matrices task	Pair Cancelation B equal to M Cancelation B lower than M ANCOVA (pt PPVT) B equal to M Flanker Task Accuracy B higher than M RTs B equal to M Frog Matrices Task Accuracy B higher than M
Carlson and Meltzoff (2008)	M B	17 12	6.25 (0.34) 6.00 (0.54)	53 33	English Spanish–English	n.r.	LSBQ (parents)	Advanced DCCS (Zelazo et al., 1996) VCR (Zelazo et al., 2002) ANT (Rueda et al., 2004) Gift Delay with Cover Simon says (Strommen, 1973) modified KRISP (Wright, 1971) Statue (Korkman et al., 1998) Delay of Gratification (Mischel et al., 1989)	All EF tasks B equal to M ANCOVA (age, verbal ability, SES) DCCS B higher than M Gift Delay with Cover, VCR, ANT (accuracy), Simon says, Delay of gratification, Statue, KRISP B equal to M Composite score for EF B better than M
Cockcroft (2016)	M B	67 53	6.81 (0.61) 6.64 (0.65)	45 47	English African–English	Africa	African as first language. Exposed to English before the age of 3. Use of both languages daily.	Forward, Backward, Counting Digit Recall Non-word Recall	All task B equal to M
Cottini et al. (2015)	M Group 1 Group 2 B Group 1 Group 2	49 25 24 55 28 27	8.2 10.4 8.3 10.2	48 42 64 67	Italian Italian–German	Italy	modified LSBQ	Global-local task Color-shape binding task	Global-local task Accuracy Monolingual Global higher than Local Bilingual Local higher than Global Incongruent, neutral trials B higher than M Global Incongruent trials B lower than M Total Interference Effect B lower than M Color-shape binding Shape-only condition accuracy B higher than M Color-only condition, combination condition B equal to M Hits B higher than M False alarms (combination condition) B higher than M
Danahy et al. (2007)	M B	50 22	10.7 (1.10) 9.9 (1.5)	56 59	English Spanish–English	n.r.	Sequential bilingual	Counting span	B equal to M ANCOVA (age) set and span score B equal to M
Dick et al. (2019)	M B-status Subgroup B-degree	2761 1740 606	9- to 10- years-old	- - -	English English–Mixed	United States	ABCD YAS (modified version of the PhenX Acculturation Measure) Bilingual status: who speak another language in addition to English. Bilingual degree: use of the non-English language frequently. Bilingual Use (continuum): how often children use the other language with friends and family	NIH Toolbox Flanker Inhibitory Control and Attention Test NIH Toolbox Stop-signal Task NIH Toolbox Dimensional Change Card Sort	Simple and multiple regressions All task B equal to M
Duñabeitia et al. (2014)	M B	252 252	10.5 (1.73) 10.5 (1.75)	54 57	Spanish Spanish–Basque	Spain Basque country	Linguistic competence questionnaire	Classic Stroop Task Numerical Stroop Task	Stroop (Classic and Numerical) RTs, accuracy, congruency and incongruity effect B equal to M
Engel de Abreu (2011)	M Kindergarten 1 grade 2 grade B Kindergarten 1 grade 2 grade	22 22	6.30 (0.23) 7.28 (0.26) 8.28 (0.26) 6.31 (0.25) 7.29 (0.26) 8.29 (0.26)	64 64	Luxembourgish Luxembourgish–Mixed	Luxembourg	Background questionnaire caregiver	Forward, Backward, Counting Digit Recall Non word repetition task	Forward, Backward, Counting Recall ANOVA, ANCOVA (expressive vocabulary) B equal to M Non word repetition task ANOVA B lower than M ANCOVA (expressive vocabulary) B equal to M
Engel de Abreu et al. (2014)	M B	33 33	8.1 (3.26) 8.2 (2.63)	51 54	Portuguese Portuguese–Luxembourgish	Portugal Gran Duchy of Luxembourg	Language and Social Background Questionnaire	Forward, Counting Digit Recall Dot Matrix task Odd-One-Out Sky Search task (Manly et al., 1999) Flanker Task	Digit and Counting Recall, Dot Matrix task, Odd-One-Out B equal to M Sky Search Task, Flanker Task B faster M
Engel de Abreu et al. (2012)	M B	40 40	8.17 (0.32) 8.25 (0.27)	50 50	Portuguese Portuguese–Luxembourgish	Portugal Gran Duchy of Luxembourg	Luxembourg Language and Background Questionnaire – Caregivers	Odd-One-Out Dot Matrix task Sky Search task (Manly et al., 1999) Flanker Task	Odd-One-Out, Dot Matrix B equal to M Flanker Task RTs B faster than M Accuracy B equal to M Sky Search Attention B faster M
Escobar et al. (2018)	M B	17 17	7.10 (3.6) 7.10 (3.5)	59 59	English English–Mixed	Australia	Language Background Questionnaire	Dimensional change card sorting task Day-Night Stroop task Verbal fluency task	DCCS, Day-Night Stroop task B equal to M Verbal fluency task n° of words B higher than M
Filippi et al. (2015)	M B	20 20	8.8 (1.2) 8.8 (1.0)	45 45	English English–Mixed	London	Parent questionnaire	Forward and backward digit span	Forward and backward digit span B equal to M
Friesen et al. (2015)	7-year-old M B 10-year-old M B	16 23 22 23	7.7 (0.3) 7.7 (0.4) 10.6 (0.5) 10.6 (0.4)	n.r.	English English–Mixed English English–Mixed	n.r.	LSBQ	Verbal fluency test	7-year-old letter and semantic condition B equal to M mean subsequent-response latency B higher than M 10-year-old semantic (n° words) B lower than M letter condition B equal to M Proportion score B lower than M
Gangopadhyay et al. (2016)	M B	42 42	9.25 (1.03) 9.38 (1.03)	52 48	English English–Spanish	Madison, Wisconsin	Parent interview	N-back Corsi blocks	N-Back, Corsi task B equal to M
Garraffa et al. (2015)	Group 1 M1 B1 Group 2 M2 B2	20 18 25 22	6.60 (0.31) 6.65 (0.32) 7.68 (0.26) 7.80 (0.47)	n.r.	Italian Italian–Sardinian	Italy	Parental Background Questionnaire Bilingual: UBILEC cumulative exposure index parameter for Italian < 3.3	Forward Digit Span Non word repetition Opposite word task DCCS	Forward Digit Span, Non word repetition B equal to M Opposite word task B slower than M B1 slower than M1 B2 faster than M2 DCCS Average score B higher than M M1 equal to M2 B2 higher than B1
Gathercole et al. (2010)	Study 1 Primary age M OEH WEH OWH Teen M OEH WEH OWH	21 36 34 36 20 41 34 36	7.11 (n.r.) 14.6 (n.r.)	n.r.	M: Welsh B: English -Welsh	North Wales	Background Questionnaire OEH: use of English ≥ 80% at home in speech to the child from birth to the present OWH: use of Welsh ≥ 80% at home in speech to the child from birth to the present	Study 1 Stroop Task English Version Stroop Task Welsh Version Study 2 Tapping task	Stroop Welsh Accuracy B equal to M RTs B equal to M Stroop English Accuracy Primary age
	Study 2 Primary age M OEH WEH OWH Teen M OEH WEH OWH	22 22 23 23 20 25 24 34	8.1 (n.r.) 14.5 (n.r.)				WEH: use of both languages (40/60%) at home in speech to the child from birth to the present		OWH lower than OEH, WEH M lower than WEH M equal to OEH Teen B equal to M RTs Primary Age B equal to M Teens M lower than B Tapping task Primary age Match condition OWH, OEH higher than M WEH equal to M WEH equal to OWH, OEH Switch condition OWH, WEH higher than OEH, M Teen Match and switch condition OWH, WEH higher than OEH, M Difference score Primary age B equal to M Teen M, OEH higher than WEH, OWH
Gathercole et al. (2014)	Study 1 5-year-old M OEH WEH OWH Primary Schoolers M OEH WEH OWH Teens M OEH WEH OWH Study 2 5-year-old M OEH WEH OWH Primary Schoolers M OEH WEH OWH Teens M OEH WEH OWH	20 16 19 16 13 20 17 14 20 28 31 35 14 20 16 21 25 22 20 29 20 24 26 34	5.4 8.2 14.9 5.5 8.0 14.9	n.r.	English Welsh–English	North Wales	Background Questionnaire OEH: use of English ≥ 80% at home in speech to the child from birth to the present OWH: use of Welsh ≥ 80% at home in speech to the child from birth to the present WEH: use of both languages (40/60%) at home in speech to the child from birth to the present	Study 1 Simon Task Study 2 modified Card sort task	Simon Age 5 Accuracy B equal to M RTs OEH slower than M OWH faster than OEH M equal to WEH Age 8 Accuracy & RTs B equal to M Teens Accuracy & RTs B equal to M Card sort task RTs B equal to M Switch cost 5-year-old, primary age B equal to M Teen OWH lower than M, WEH
Hartanto et al. (2019)	Wave 1 M B Wave 2 M B Wave 3 M B Wave 4 M B	10,133 1,155 10,246 1,372 2,331 541 7,012 1,091	5.58 (0.36) 5.63 (0.37) 6.06 (0.36) 6.13 (0.37) 6.55 (0.36) 6.59 (0.36) 7.05 (0.35) 7.13 (0.37)	50 49 50 48 50 47 50 49	M: English; B: English–Mixed	United States	Sufficient English skills; speak a language other than English at home.	Dimension Change Card Sort Task Numbers Reversed Subtest	Dimension Change Card Sort Task B higher than M Beneficial effect of bilingualism Yes low-SES families No middle/high-SES families Numbers Reversed Subtest Wave 3 B equal to M Wave 4 B higher than M High SES Wave 3 and 4 B equal to M Low SES Wave 3 B equal to M Wave 4 B higher than M
Jaekel et al. (2019)	M B	95 242	9.3 (3.1) 9.7 (2.1)	49.5 58.4	German German–Turkish	Rurh area, Germany	Parents or grandparents born in Turkey	Hearts and Flowers task Digit Span Backward	Hearts and Flower task B equal to M Digit span backward B lower than M
Jalali-Moghadam and Kormi-Nouri (2015)	M B	59 45	10.39 (0.96) 10.62 (1.11)	54 64	Swedish Iranian–Swedish	Sweden	Language History Questionnaire–children	Stroop Task Concentration task Tower of Hanoi	Stroop, Concentration task, Tower of Hanoi B equal to M
Janus and Bialystok (2018)	M B	48 45	9.3 (0.6) 9.4 (0.5)	37 44	English English–Mixed	-	LSBQ	Emotional Face N-Back Task	Target trials Accuracy B higher than M RTs 1-back B equal to M 2-back B slower than M Nontarget trials Accuracy B equal to M RTs B slower than M
Kapa and Colombo (2013)	M EB LB	22 21 36	9.81 (2.33) 9.13 (2.42) 9.88 (2.32)	45 57 64	English Spanish–English English–Spanish	U.S.	Parent Questionnaire	Forward Digit Span ANT (Rueda et al., 2004)	Forward Digit Span B equal to M ANT ANCOVA (age, vocabulary) RTs EB faster than M M equal to LB EB equal to LB Accuracy, alerting, orienting, conflict B equal to M
Krizman et al. (2016)	M B	32 30	14.5 (0.3) 14.7 (0.4)	55 45	English Spanish–English	Chicago, Illinois	Language Experience and Proficiency Questionnaire Parental report of the child's language abilities	Integrated Visual and Auditory Continuous Performance Test	B better than M
Ladas et al. (2015)	Study 1 M B Study 2 M B	24 26 32 28	9.43 (1.46) 9.28 (1.57) 6.44 (0.82) 6.77 (0.56)	75 38 56 54	Greek Albanian–Greek Greek Albanian–Greek	Greece	Albanian ethnicity, spoke Albanian and Greek approximately equally in everyday life, and had been exposed to both languages from 2 years of age or earlier	Study 1 ANT (Rueda et al., 2004) Study 2 ANTI (Callejas et al., 2004)	ANT B equal to M ANTI B equal to M
Leikin and Tovli (2014)	All M B	31 16 15	5.99 (0.3) n.r. n.r.	n.r.	Hebrew Russian–Hebrew	Israel	Parent Questionnaire (Leikin, 2013) Russian/Hebrew-speaking raters	Listening Recall	Correct word B more than M Correct sequences B equal to M Total B higher than M
Mohades et al. (2014)	M L2 learners B	14 18 19	9.58 (1.00) 9.50 (0.83) 9.42 (0.92)	50 50 53	French or Dutch French or Dutch French or Dutch–Romance or Germanic languages	Belgium	Both languages from birth	Simon task Numerical Stroop task	Simon task RTs, ACC B equal to M Congruency effect B higher than M Stroop task RTs, ACC B equal to M Congruency effect B higher than M
Morales et al. (2013)	Study 1 5-year-old M B Study 2 5-year-old See Study 1 7-year-old M B	56 29 27 69 34 35	5.42 (0.45) n.r. n.r. 6.92 (0.23) n.r. n.r.	41 59 47 49	English English–Mixed English English–Mixed	n.r.	Language History Questionnaire -parents	Study 1 Picture Task Study 2 Frog Matrices Task	Picture Task RTs B faster M Frog Matrices Task Proportion Score B higher than M
Nayak et al. (2020)	M B	61 51	6.98 (0.57) 6.85 (0.62)	47 51	English English–Mixed	-	Children dominant in English and exposed to an L2 for > 20% of time. Children exposed to South Asian languages were excluded.	Animal Size Stroop Task	ANCOVA (age, SES) Stroop Effect (RTs) B equal to M ANCOVA (SES) Stroop Effect (ACC) B equal to M
Park et al. (2018)	M Year 1 Year 2 B Year 1 Year 2	41 41	9.39 (0.98) 10.40 (0.97) 9.42 (1.03) 10.45 (1.04)	51 44	English English–Spanish	n.r.	Parent Questionnaire	Flanker Task Corsi Blocks DCCS	Flanker Task Inhibition skills Year 1 B equal to M Year 2 B higher than M Corsi Blocks B equal to M DCCS Shifting cost, switching cost B equal to M Mixing cost B lower than M
Poarch and Bialystok (2015)	M B	60 60	9.5 (1.0) 9.4 (0.8)	52 47	English English–Mixed	n.r.	LSBQ	modified Flanker Task	RTs Incongruent trials B faster than M Conflict effect B better than M
Poarch and van Hell (2012)	M B	20 18	7.1 (0.5) 6.8 (0.7)	45 61	German German–English	Germany	Parent Questionnaire	Simon Task (Bialystok et al., 2004; Simon and Rudell, 1967)	Simon RTs, Accuracy, Simon Effect B equal to M
Raudszus et al. (2018)	M B	76 102	9.99 (0.44) 9.95 (0.43)	n.r. n.r.	Dutch Dutch–Mixed	The Netherlands	interview to children	Simon Task (Simon and Wolf, 1963) Backward Digit Span	Simon Effect B equal to M Backward Digit Span B equal to M
Ross and Melinger (2017)	Study 1 M B Study 2 M B	45 54 21 49	7.71 (0.63) 7.67 (0.58) 7.39 (0.12) 7.69 (0.08)	n.r. n.r. n.r. n.r.	English English–Mixed English English–Mixed	England Scotland England Scotland	Language Background Questionnaire	Study 1 Simon Task (Simon and Wolf, 1963) modified Flanker Task Study 2 BCST	Simon Accuracy B higher than M RTs, Simon Effect B equal to M Flanker task B equal to M
									BCST n° errors B higher than M preservative errors, n° of trials needed to achieve a category, RTs for correct responses B equal to M
Schröter and Schroeder (2017)	M B	64 34	12 (0.84) 12 (1.00)	58 41	German Mixed–German	Germany	Parent Questionnaire	Stroop Task – Heathcote et al., 1991 Listening Recall Opposite Worlds task	Stroop B equal to M Listening Recall B equal to M Opposite Worlds task B equal to M
Struys et al. (2018)	YM YB OM OB	29 30 29 29	6.7 (0.3) 6.6 (0.3) 11.6 (0.3) 11.7 (0.3)	41 57 55 72	Dutch Dutch–Mixed	Belgium	Questionnaire parents	Simon task Flanker task	Simon task Simon effect YB higher than YM OB equal to OM Accuracy B equal to M Speed-Accuracy Trade-off No YM, OM Yes YB (incongruent trials), OB (global performance, incongruent trials) Flanker task Flanker effect YB equal to YM OB lower than OM Accuracy B equal to M Speed-Accuracy Trade-off No YM, YB, OM Yes OB (global performance, incongruent trials)
Veenstra et al. (2018)	M B	44 45	11.1 (0.58) 11.1 (0.58)	57 62	Dutch French–Dutch	the Netherlands Belgium	modified version of Alberta Language Environment Questionnaire	Forward and Backward Digit Span Corsi Blocks Color-shape task switching ANT (Rueda et al., 2004)	All task ANCOVA (age, SES, vocabulary size) B equal to M
Yang and Yang (2016)	M B	31 32	5.10 (0.60) 5.10 (0.55)	26 41	English Korean–English	New York, New Jersey	Parent Questionnaire	ANT (Rueda et al., 2004)	RTs B faster than M Accuracy B higher than M Alerting, orienting, conflict B equal to M
Zeng et al. (2019)	M B	17 20	8.3 (1.5) 8.3 (1.2)	60 60	Australian English Australian English–Mixed	-	LSBQ (parents)	Letter and category Verbal Fluency Task Simon Arrow Task	ANCOVA (age) Category VFT B equal to M Letter VFT B higher than M Simon task general ACC B higher than M Neutral, opposite, conflict condition B higher than M Conflict condition B higher than M RTs B equal to M

*N, number of participants; SD, standard deviation; M, monolingual participants; B, bilingual participants; TMT, Trial Making Test; n.r., not reported; ANT, Attention Network Test; RT, reaction time; ACC, accuracy; BCE, Chinese-English bilingual; BFE, French-English bilingual; BSE, Spanish-English bilingual; LSBQ, Language and Social Background Questionnaire; VCR, Visually Cued Recall Task; DCCS, Dimensional Change Card Sort Task; PI, Proactive Interference Task; BC, Canadian bilingual; BI, Indian bilingual; BFD, Frisian-Dutch bilingual; BLD, Limburgish-Dutch bilingual; BPD, Polish-Dutch bilingual; PaBiQ, Questionnaire for parents of bilingual children; WCM, working class monolingual; MCM, middle class monolingual; WCB, working class bilingual; MCB, middle class bilingual; PPVT, Peabody Picture Vocabulary Test; KRISP, Kansas Reflection/Impulsivity scale; SES, socioeconomic status; OEH, only English at home; WEH Welsh and English at home; OWH, only Welsh at home; ANTI, Attention Network Test for Interaction; BCST, Berg Card Sorting Test*.

a *subsample 31 monolingual vs. 19 bilingual*.

**Table 2:** Bilingual participants' characteristics in the selected studies.

**Authors**	**Characteristic of bilingual participants**							**Evaluation of language abilities**	**Socioeconomic status (SES)**
		**Language pair**	**AoA**	**School language**	**Home language**	**Home language – Parents**	**Home language – child**		
Abdelgafar and Moawad (2015)	B	Arabic – English	n.r.	English + Arabic	50 % each language	n.r.	n.r.	Arabic reading test B equal to M Arabic reading abilities equal to English reading abilities	B equal to M Mother and father education: high education Income: more than 4,000 $
Antón et al. (2014)	B	Spanish–Basque	Spanish 0.58 (0.77) Basque 2.23 (1.07)	50% Spanish, 50% Basque	n.r.	n.r.	n.r.	Parents' subjective rating Spanish 8.65 (1.17) out of 10 Basque 5.96 (1.63) out of 10 Reading skills -teachers B equal to M	Income, Parents' education, parents' work situation B equal to M Income^i^ Bilingual 1.74 (0.93) Monolingual 1.89 (0.89) Parents' years of education Bilingual 14.50 (2.31) Monolingual 14.13 (2.50) Parents' work situation^ii^ Bilingual 1.94 (0.26) Monolingual 1.95 (0.24)
Arizmendi et al. (2018)	B	English–Spanish	n.r.	n.r.	only Spanish or Spanish and English	only Spanish or Spanish and English	only Spanish or Spanish and English	CELF-4 English and Spanish B lower than M EVT-2 B lower than M WRMT B lower than M EOWPVT – Bilingual version	Maternal level of education B lower than M
Barac and Bialystok (2012)	BCEBFEBSE	Chinese–EnglishFrench–EnglishSpanish–English	n.r.	EnglishFrenchEnglish	n.r.	3.9 (0.9)^iii^3.2 (0.9)^iii^3.5 (1.0)^iii^	2.9 (0.9)^iii^3.0 (1.0)^iii^2.7 (0.9)^iii^	PPVT-III M, BSE higher than BCE BCE equal to BFE CELF-4 M, BSE higher than BFE BCE equal to M, BSE, BFE Wugs test BSE higher than M, BCE, BFE	Parents' years of education B equal to
Barac et al. (2016)	B	English–Mixed	36 % simultaneous bilinguals	n.r.	n.r.	3.16 (1.37)^iii^	2.72 (1.21)^iii^	Vocabulary subtest from WPPSI-III B lower than M	Level of maternal education B equal to M
Bialystok (1999)	B	Cantonese/Mandarin–English	n.r.	English	Cantonese/Mandarin	n.r.	n.r.	PPVT-R B equal to M	No information
Bialystok (2010)	B1 B2 B3	English–Mixed English–Mixed English–Mixed	n.r. n.r. n.r.	English English English	non-English language non-English language non-English language	2.62 (1.2)^iii^ 2.40 (0.6)^iii^ 1.00 (0.9)^iii^	3.15 (0.8)^iii^ 3.20 (0.4)^iii^ 2.00 (0.7)^iii^	PPVT-III B equal to M	Middle-class neighborhood. Parents worked primarily in professional and management occupations.
Bialystok (2011)	B	English–Mixed	n.r.	n.r.	2.4 (0.6)^iii^	n.r.	n.r.	PPVT-III B equal to M	B equal to M Middle class
Bialystok and Feng (2009)	B	English–Mixed	n.r.	English	non-English language	3.5 (1.1)^iii^	2.3 (0.8)^iii^	PPVT-III B lower than M	No information
Bialystok and Viswanathan (2009)	BC BI	English–Mixed English–Tamil/Telugu	n.r.	EnglishEnglish	54% English 47% English	n.r.	n.r.	PPVT-III B lower than M BI equal to BC	Middle-class
Blom et al. (2017)	BFD BLD BPD	Frisian–Dutch Limburgish–Dutch Polish–Dutch	30% Dutch* 70% non-Dutch* 40% Dutch* 59% non-Dutch* 43% Dutch* 54% non-Dutch* *before the age of four	n.r.	n.r.	31% Dutch 68% non-Dutch 42% Dutch 56% non-Dutch 37% Dutch 61% non-Dutch	n.r.	PPVT – Dutch version BPD lower than M, BFD, BLD	Average education level of both parents B equal to M BFD, BLD all born in the Netherlands. BPD 70 % were born in the Netherlands. All children had lived in the Netherlands at least for 2 years
Blom et al. (2014)	B	Turkish–Dutch	n.r.	n.r.	n.r.	48% Turkish; 45% Turkish and Dutch; 6% n.r.	n.r.	Toets Tweetaligheid (Test for Bilingualism) –Dutch and Turkish 15 items from Taaltoets Alle Kinderen (Language Test for All Children)–Dutch B lower than M	Average education level of both parents B lower than M
Bonifacci et al. (2011)	B	Italian–Mixed	n.r.	Italian	n.r.	n.r.	n.r.	No test	No information
Bosman and Janssen (2017)	B	Turkish–Dutch 65% Turkish as L1	80% exposed to Dutch before the age of three	Dutch	Turkish	n.r.	n.r.	Reynell test for language comprehension B lower than M Dutch and Turkish sentence imitation task B lower than M Dutch higher than Turkish	B equal to M low-SES All Turkish-Dutch children were born in the Netherlands
Buac et al. (2016)	B	Spanish–English Spanish as L1	Simultaneous and sequential bilinguals	48% attended a Spanish-English dual immersion program	n.r.	59% Spanish, 24% English, 17% Both	n.r.	PPVT-III–English Picture vocabulary subtest – English expressive vocabulary B lower than M TVIP – Spanish	Caregiver's total number years of education B lower than M
Calvo and Bialystok (2014)	B	English–Mixed	n.r.	n.r.	2.6 (0.6)^iii^	n.r.	n.r.	PPVT-III B lower than M WC lower than MC	MC: mother had completed at least some post-secondary education WC: high school or less education
Carlson and Meltzoff (2008)	B	Spanish–Mixed	Exposure to English and Spanish from birth	n.r.	n.r.	n.r.	n.r.	One-word picture vocabulary test- Spanish/English bilingual edition B lower than M	Maternal education, family income, amount of time parents read to children B lower than M
Cockcroft (2016)	B	African–English African as L1	Exposed to English before the age of three	English	n.r.	n.r.	n.r.	BPVS-II – receptive vocabulary BNT – expressive vocabulary B lower than M	Living Standard Measure, occupational status, educational level B equal to M
Cottini et al. (2015)	B	Italian–German	n.r.	German	n.r.	n.r.	n.r.	LSBQ-parents and teachers Fluency G: 3.78, I: 3.67 Reading G: 4.00, I: 3.60 Writing G: 3.46, I: 3.51 Comprehension G: 3.71, I: 3.49 Grammar G: 3.59, I: 3.43	No information
Danahy et al. (2007)	B	Spanish–English Spanish as L1	4-8 years of English experience	English	n.r.	n.r.	n.r.	CELF English and Spanish	No information
Dick et al. (2019)	B	English–Mixed	Second language before 10 years of age	n.r.	n.r.	n.r.	n.r.	NIH Toolbox Vocabulary test Children self-report (English) Excellent: 71.2 %; good: 25.7 %; fair: 2.9 %; poor: 0.1 %.	Highest household education, household marriage status, highest household income, race/ethnicity n.r.
Duñabeitia et al. (2014)	B	Spanish–Basque	Spanish 0.75 (0.89) Basque 2.27 (1.11)	50% Spanish, 50% Basque	n.r.	n.r.	n.r.	Linguistic competence questionnaire Spanish: 8.68 (1.23) Basque: 6.10 (1.75)	All participants lived in (and were originally from) the same country
Engel de Abreu (2011)	B	Luxembourgish–Mixed	Exposure from birth to languages	Luxembourgish	n.r.	n.r.	n.r.	EOWPVT TROG-2 B lower than M	Parental education B equal to M
Engel de Abreu et al. (2014)	B	Portuguese–Luxembourgish Portuguese as L1	n.r.	Luxembourgish	Portuguese	n.r.	n.r.	EOWPVT PPVT-4–Portuguese and Luxembourgish TROG-2 – Portuguese, Luxembourgish B lower than M	International Socio-Economic Index of Occupational Status. Highest occupational level of either caregivers B equal to M 70% born in Luxembour 30% emigrated to Luxembourg before the age of 3
Engel de Abreu et al. (2012)	B	Portuguese–Luxembourgish Portuguese as L1	n.r.	Luxembourgish	Portuguese	n.r.	n.r.	EOWPVT B lower than M	Low-SES families International Socio-Economic Index of Occupational Status B equal to M Caregiver Education B lower than M 25% first-generation immigrants (immigrated before the age of 3) 75% second-generation immigrant
Escobar et al. (2018)	B	English–Mixed	L1 and L2 from birth	English	At least one parent use of non-English language	n.r.	n.r.	PPVT-4 B equal to M L2 Parental rating	Parental education B equal to M
Filippi et al. (2015)	B	English–Mixed	Exposed to English from birth/ before the age of three	English	non-English language	n.r.	n.r.	BPVS-II B equal to M	Parent's level of education: university degree or higher B equal to M
Friesen et al. (2015)	7-year-old 10-year-old	English–Mixed English–Mixed	L2: 3.2 (1.5) L2: 3.8 (2.3)	n.r. n.r.	non-English non-English	n.r. n.r.	n.r. n.r.	PPVT-III 7-year-old B equal to M 10-years-old B lower than M	No information
Gangopadhyay et al. (2016)	B	English–Spanish	English: 8.02 (12.66) months Spanish: 1.57 (6.59) months	English: 55%; Spanish: 12%; both: 33%	n.r.	n.r.	n.r.	CELF-4 B lower than M Expressive vocabulary English higher than Spanish Receptive vocabulary English equal to Spanish	Maternal years of education B lower than M
Garraffa et al. (2015)	B	Italian–Sardinian	n.r.	Italian	Sardinian	n.r.	n.r.	PPVT-4 B equal to M COMPRENDO test B equal to M	No information
Gathercole et al. (2010)	OEH OWH WEH	English–Welsh English–Welsh English–Welsh	n.r.	Welsh	80% use of English 80% use of Welsh use of both languages	n.r.	n.r.	BPVS Prawf Geirfa Cymraeg	Parents' education and professions
Gathercole et al. (2014)	OEH OWH WEH	English–Welsh English–Welsh English–Welsh	n.r.	Welsh	80% use of English 80% use of Welsh use of both languages	n.r.	n.r.	Vocabulary and receptive grammatical tests	No information
Hartanto et al. (2019)	B	English–Mixed	n.r.	English	non-English	n.r.	n.r.	Preschool Language Assessment Scales B lower than M	Household income, parental education, parental occupation prestige, SES composite scores B lower than M
Jaekel et al. (2019)	B	German–Turkish	n.r.	German	n.r.	Turkish Family Environment Index Score n.r.	Turkish Family Environment Index Score n.r.	Turkish vocabulary modified version of PPVT-4 German vocabulary modified version of EOWPVT	All immigrant children of second or third generation. Maternal and paternal education
Jalali-Moghadam and Kormi-Nouri (2015)	B	Iranian–Swedish	n.r.	Swedish – 1-hour Farsi	Farsi	n.r.	n.r.	No test	Parent's occupation and education B equal to M
Janus and Bialystok, 2018	B	English–Mixed	n.r.	English	non-English	n.r.	n.r.	PPVT B equal to M	Parental education B equal to M
Kapa and Colombo (2013)	EB LB	Spanish–EnglishSpanish–English	EA: Acquisition of both language between 1-3 years of age; LB: acquisition of Spanish before the age of three and English after 3 years.	English	n.r.	n.r.	n.r.	PPVT-III B lower than M EB equal to LB TVIP – Spanish EB equal to LB	Parents' education B lower than M EB equal to LB
Krizman et al. (2016)	B	Spanish–English 55% Spanish as L1	Spanish 2.1 (1.7) English 3 (1.8)	n.r.	n.r.	n.r.	n.r.	Self-rated English proficiency B equal to M	Education level of mother low-SES: high school or less high-SES: college or higher
Ladas et al. (2015)	B	Albanian–Greek Albanian as L1	Exposure to both languages from two years of age or earlier	Greek	n.r.	n.r.	n.r.	Vocabulary subtest WISC – Albanian and Greek Greek B equal to M	All low-SES B equal to M *S*econd-generation Albanian immigrants
Leikin and Tovli (2014)	B	Russian–Hebrew	n.r.	n.r.	Russian	n.r.	n.r.	Russian/Hebrew-speaking raters: level of Hebrew almost equivalent to the level of Russian	Parents' education level B equal to M Russian immigrants from the former USSR
Mohades et al. (2014)	B	L1: Dutch or French; L2: Romance or Germanic languages	L1 and L2 from birth	n.r.	n.r.	n.r.	n.r.	Verbal auditory discrimination, verbal fluency tests, listening-comprehension, sentence-construction tests in both languages	B equal to M
Morales et al. (2013)	B	English–Mixed	n.r.	English	non-English	3.5 (1.0)^iii^	2.5 (1.1)^iii^	PPVT-III B lower than M	Middle-class community. All parents had at least college-level diploma
Nayak et al. (2020)	B	English–Mixed	n.r.	n.r.	n.r.	n.r.	n.r.	No test	Parent Occupational Prestige, Parent Education, Income-to-Needs Ratio, Annual Income B equal to M
Park et al. (2018)	B	English–Spanish	Exposure to both languages before the age of three	English	more non-English language	n.r.	n.r.	CELF-4 – English B lower than M	Year of maternal education B lower than M
Poarch and Bialystok (2015)	B	English–Mixed	n.r.	English	non-English language	3.0 (1.2)	n.r.	PPVT-III B equal to M	
Poarch and van Hell (2012)	B	German–English	Exposure to both languages from birth	German-English	n.r.	n.r.	n.r.	TROG – English and German English equal to German	Parents' education levels B equal to M
Raudszus et al. (2018)	B	Dutch–Mixed	n.r.	Dutch	non-Dutch	n.r.	n.r.	Language Test for Minority Children Grades 4–6 Dutch PPVT-III Passive Vocabulary subtest of the Toets Tweetaligheid [Diagnostic Test of Bilingualism]	No information
Ross and Melinger (2017)	B	English–Mixed	n.r.	n.r.	n.r.	n.r.	n.r.	BPVT-II B lower than M	Low to middle range for SES of deprivation
Schröter and Schroeder (2017)	B	German–Mixed non-German language as L1	German acquired before the age of six	n.r.	79% both, 21 % only native language	n.r.	n.r.	CFT 20-R – vocabulary listening comprehension B lower than M	Children's cultural resources, communicative practices, children's reading pleasure and reading motivation B equal to M
Struys et al. (2018)	YBOB	Dutch–Mixed	YB: L1 from birth; L2: 0.8 (0.8) OB: L1 from birth; L2: 0.7 (0.8)	n.r.	n.r.	n.r.	n.r.	L1 parental rating B lower than M L2 parental rating	Composite score of parents' education levels B equal to M
Veenstra et al. (2018)	B	French–Dutch	n.r.	Dutch	French	n.r.	n.r.	PPVT-III–Dutch CELF-4 B lower than M	B higher than M
Yang and Yang (2016)	B	Korean–English Korean as L1	Sequential bilingual	Korean	English	n.r.	n.r.	PPVT – III B lower than M	Parents' education level minimum college education middle class neighbor Second-generation Korean immigrant
Zeng et al. (2019)	B	Australian English–Mixed	One child from birth both languages; L1 from birth, L2 2.95 (0.85)	English	Both languages	n.r.	n.r.	L1 PPVT-IV, EVT B equal to M L2 Parental report	No information

*B, bilingual participants; M, monolingual participants; AoA, age of acquisition; CELF, Clinical Evaluation of language fundamentals; EVT, Expressive Vocabulary Test; WRMT, Woodcock Reading Mastery Test, Paragraph Comprehension Subtest; EOWPVT, Expressive One Word Picture Vocabulary Test; BCE, Chinese-English bilingual; BFE, French-English bilingual; BSE, Spanish-English bilingual; PPVT, Peabody Picture Vocabulary Test; WPPSI, Wechsler Preschool and Primary Scale of Intelligence; BC, Canadian bilingual; BI, Indian bilingual; BLD, Limburgish-Dutch bilingual; BFD, Frisian-Dutch bilingual; BPD, Polish-Dutch bilingual; TVIP, Test de Vocabulario en Imagenes Peabody, MC, middle class; WC, working class; BPVS, British Picture Vocabulary Test; BNT, Boston Naming Test; NIH, National Institutes of Health; OEH, only English at home; WEH Welsh and English at home; OWH, only Welsh at home; EB, early-bilingual; LB, later bilingual; TROG, Test for reception of Grammar*.

i *Category 1: >3000€/month; Category 2: 2001–3000 €; Category 3: 1601–2000 €; Category 4: 1201–1600 €; Category 5: 750–1200 €; Category 6: <750 €*.

ii *Category 1: neither works; Category 2: only one of them works; Category 3: both works*.

iii *1: exclusive use of English, 5: exclusive use of non-English language*.

## Results

### Selection of Studies

**Figure 1:**
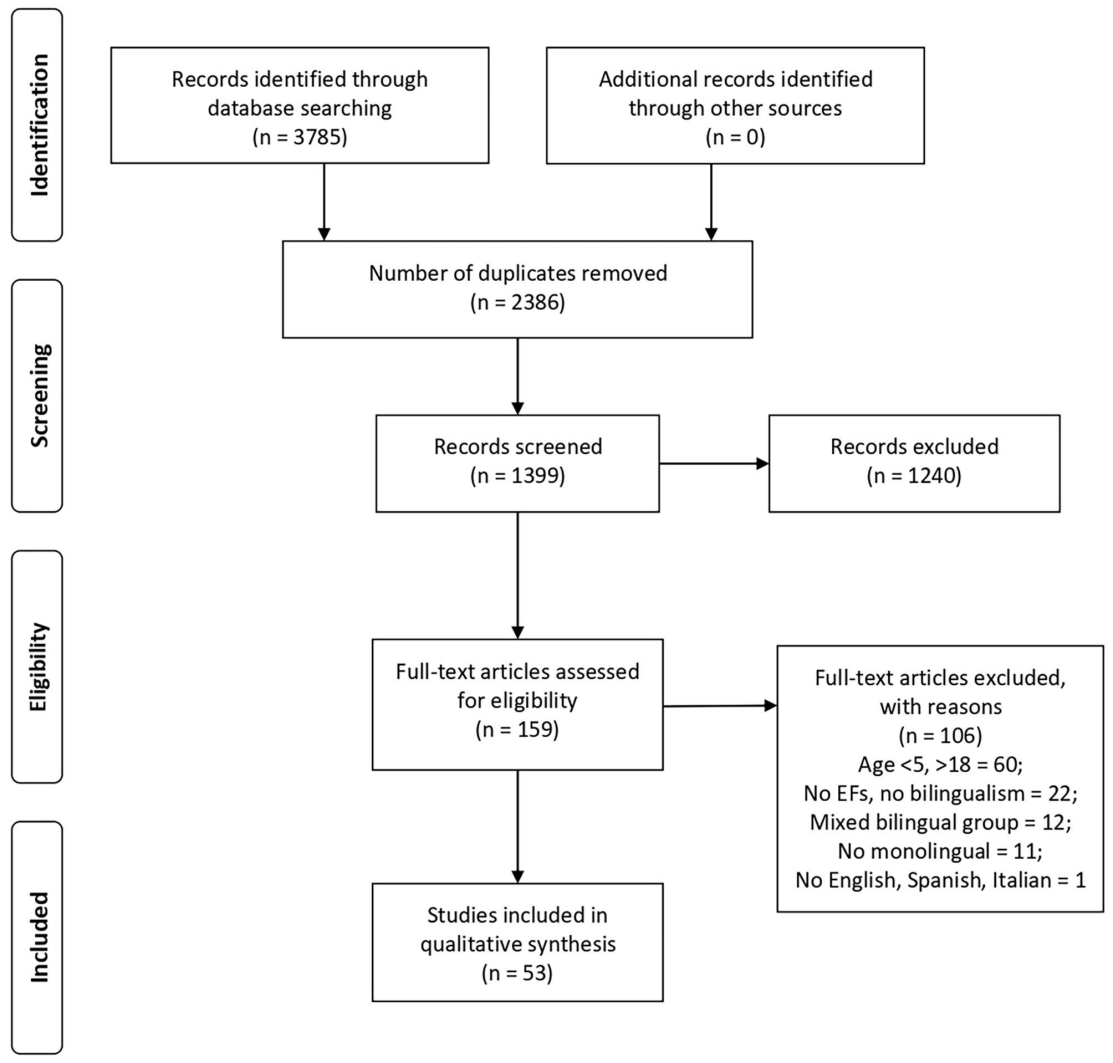
Studies selection flow diagram (PRISMA flow chart).

The flowchart (Figure 1) shows the number of studies identified from the databases and the other sources, the number of studies examined by the authors, and assessed for eligibility. The reasons for exclusion are reported.

### Results of the Selected Studies

Of the 53 studies identified, 24 were conducted in Europe, 10 in America, two in Asia, one in Africa, one in Australia, and 14 did not report the country. Bialystok and Viswanathan (2009) included participants from two different continents (America and Asia).

Twenty-seven studies included bilingual participants who knew a specific language pair while in 23 studies, bilinguals spoke a common language plus another language. Bialystok and Viswanathan (2009) included two groups of bilingual participants, one speaking a specific language pair, the other speaking different languages. Two studies (Barac and Bialystok, 2012; Blom et al., 2017) included distinct groups of bilingual participants with different linguistic backgrounds to check if the type of language known, influenced the results.

In most studies, information on the participants' linguistic background was collected through interviews or questionnaires made to their caregivers. In two studies, the information was collected by directly interviewing the participants (Jalali-Moghadam and Kormi-Nouri, 2015; Raudszus et al., 2018). The analyzed studies reported different definitions of bilingualism; some of these definitions are based on the assessment of the competences in the two languages; others are founded on the age of acquisition of the two languages. Twenty-five studies reported information on the time of acquisition of the second language (e.g., type of bilingualism, the age range in which the languages were learned), but only 12 studies indicated the age of acquisition. Most of the studies did not indicate the language context in which the children were immersed, and only eight studies defined the language used at home by parents and children. Forty-five studies assessed the participants' language skills using both tests and self-report questionnaires or interviews. In twenty-four studies were assessed both languages known by the bilingual participants. In three studies (Escobar et al., 2018; Dick et al., 2019; Zeng et al., 2019), objective assessments and self-report questionnaires were used. The use of both tools allows investigating both language proficiency (tests) and language use (self-report), two aspects that can contribute to a better description of the bilingual experience (Luk and Bialystok, 2013). Twenty-four studies reported a reduced vocabulary for bilinguals compared to monolinguals considering only the groups' common language. In three studies, no assessment of the participants' language skills was conducted. Many of the studies provided information on socioeconomic status, and the most used as an indicator of SES the educational level of parents. In nine studies, the group of bilinguals had a lower socioeconomic status than monolinguals. In Veenstra et al. (2018), the bilinguals had a higher socioeconomic status than monolinguals. Nine studies did not report information on the SES (see Table 2).

#### Bilingualism and Attention (n = 11)

Eleven studies examined the effect of bilingualism on attention. Three studies (Engel de Abreu et al., 2012, 2014; Blom et al., 2017) used the Sky Search task of the Test of Everyday Attention for Children (Manly et al., 1999) to assess selective attention. Participants were asked to identify pairs of identical pictures on a sheet of paper while ignoring the presence of distracting stimuli. In all studies, bilingual participants took less time to solve the task compared to monolinguals.

Calvo and Bialystok (2014) used the Pair Cancellation Subtest of the Woodcock-Johnson Tests of Cognitive Abilities-III (Woodcock et al., 2001) to assess non-verbal visual attention and the cancellation subtest of Wechsler Intelligence Scale for Children-Fourth Edition (WISC-IV; Wechsler, 2003) to assess verbal-visual attention. In the task with verbal stimuli, bilinguals performed significantly worse than monolinguals, while in the task with non-verbal stimuli, no differences emerged between the two groups.

Seven studies (Carlson and Meltzoff, 2008; Kapa and Colombo, 2013; Antón et al., 2014; Ladas et al., 2015; Barac et al., 2016; Yang and Yang, 2016; Veenstra et al., 2018) used the child-friendly version of the Attentional Network Task proposed by Rueda et al. (2004) to assess the three attentional networks (alerting, orienting, and executive control).

In Ladas et al. (2015), the participants also carried out the Attentional Network Task for Interaction (Callejas et al., 2004). Four studies (Carlson and Meltzoff, 2008; Antón et al., 2014; Ladas et al., 2015; Veenstra et al., 2018) found no significant difference in performance between monolinguals and bilinguals. In Yang and Yang (2016), bilingual children were globally faster and more accurate than monolingual children. No differences were found in the three attention indexes (alerting, orienting, and executive control).

The authors also calculated the global inverse efficiency scores by dividing the mean reaction times by accuracy percentage. This index indicated an advantage for the bilingual group over the monolingual group. In Barac et al. (2016), no significant differences in RTs or attentional indexes emerged between bilinguals and monolinguals. In Kapa and Colombo (2013), both reaction times and the percentage of accuracy were analyzed by using age and vocabulary as covariates. For reaction times, the early bilingual group (i.e., children who learned both languages before the age of three) was significantly faster than the monolingual group. At the same time, no significant differences emerged between the later bilingual group (i.e., children who learned Spanish before the age of three and English after three) and the monolingual group. The two bilingual groups did not differ between them. No significant differences were found between the three groups in the percentage of accuracy and the attentional indexes.

#### Bilingualism and Visual Working Memory (n = 17)

Four studies (Bialystok and Viswanathan, 2009; Gangopadhyay et al., 2016; Park et al., 2018; Veenstra et al., 2018) used the Corsi blocks task to assess visuospatial working memory. No significant differences emerged between the performance of monolinguals and bilinguals. Four studies (Engel de Abreu et al., 2012, 2014; Blom et al., 2014, 2017) used a modified version of this task, the Dot Matrix Task, and again no significant differences between the two groups' performance were found. In the study of Blom et al. (2014) to verify whether age, socioeconomic status, defined as the average education level of both parents, and vocabulary size, influenced the results, these variables were used as covariates in the statistical analysis and participants were divided into two age groups. Results showed that bilinguals at 6 years had a better performance than monolinguals. Two studies (Morales et al., 2013; Calvo and Bialystok, 2014) used a child-friendly version of the Corsi blocks task, the Frog Matrices Task. In Calvo and Bialystok (2014), bilinguals were more accurate than monolinguals. In Morales et al. (2013), bilinguals showed a higher proportion score (calculated as the number of remembered elements divided by the total number of elements) than monolinguals in the sequential condition. In the less demanding condition, i.e., the simultaneous condition, no significant differences emerged between the two groups.

Three studies (Gangopadhyay et al., 2016; Arizmendi et al., 2018; Janus and Bialystok, 2018) used the N-back task to assess non-verbal working memory. In Gangopadhyay et al. (2016), no significant differences were found between bilinguals and monolinguals. Arizmendi's et al. (2018) study used two N-back tasks (i.e., N-back Auditory task and N-back Visual task), and monolinguals solved the tasks more efficiently than bilinguals. In Janus and Bialystok (2018), who used a modified version with emotional stimuli, bilinguals were more accurate than monolinguals when they had to indicate that the target was the same as in the previous trial (target trial) than when it was not (non-target trial). Furthermore, bilinguals had slower reaction times than monolinguals when a target trial (2-back condition) or a no target trial was presented (1-back and 2-back conditions).

Three studies (Engel de Abreu et al., 2012, 2014; Blom et al., 2014) used the Odd-One-Out task. No significant differences were found in any of the studies.

Jalali-Moghadam and Kormi-Nouri (2015) used the Concentration task (Schumann-Hengseler, 1996) and the Tower of Hanoi (Welsh, 1991) and no significant differences emerged between bilingual and monolingual participants.

Morales et al. (2013) used the Picture Task. Bilinguals solved the task more efficiently with faster reaction times in all conditions. Bilinguals had the same accuracy score in congruent and incongruent trials, while monolinguals were negatively affected by the incongruent condition.

Two studies (Bialystok, 1999; Carlson and Meltzoff, 2008) used the Visually Cued Recall task (Zelazo et al., 1997) and did not find differences between bilinguals and monolinguals.

Bonifacci et al. (2011) used two experimental tasks to assess visual working memory in which participants were required to indicate whether a target stimulus appeared within a string of stimuli. Numerical and unknown alphabetical symbols were used as stimuli. There were no significant differences between the performance of the two groups. Cottini et al. (2015) used the Color-Shape binding task (adapted from Allen et al., 2006), bilinguals were more accurate than monolinguals only in the shape condition, while there were no differences in the color condition and the combination of the two conditions. Furthermore, bilinguals presented more false alarms than monolinguals only in the combination condition.

#### Bilingualism and Verbal Working Memory (n = 21)

Four studies used the listening recall task to assess working memory. In Leikin and Tovli (2014), participants had to complete sentences with the missing word, and then they have to recall the complete list of words used (Shani et al., 2005). In two studies (Buac et al., 2016; Schröter and Schroeder, 2017), participants had to judge whether the sentences were true or false, and then remember the last word (Daneman and Carpenter, 1980). In Bosman and Janssen (2017), a modified version of this task was adopted in which participants were required to remember the first word because, in the participants' language, the last word of the sentence was always a verb. Within these studies, only Leikin and Tovli (2014) found a significant difference between groups, with bilinguals who named more correct words than monolinguals. The number of the correct sequences (i.e., the number of correct orders of the words) was the same in the two groups. In Bosman and Janssen (2017), bilingual children's performance was worse than that of monolinguals. In two studies (Buac et al., 2016; Schröter and Schroeder, 2017), no significant differences emerged.

Bialystok and Feng (2009) used the Proactive Interference Task, and no significant differences in the performance of the two groups were found.

Eighteen studies (Danahy et al., 2007; Bialystok and Feng, 2009; Bialystok and Viswanathan, 2009; Bialystok, 2010; Engel de Abreu, 2011; Kapa and Colombo, 2013; Blom et al., 2014, 2017; Engel de Abreu et al., 2014; Filippi et al., 2015; Garraffa et al., 2015; Buac et al., 2016; Cockcroft, 2016; Bosman and Janssen, 2017; Raudszus et al., 2018; Veenstra et al., 2018; Hartanto et al., 2019; Jaekel et al., 2019) evaluated working memory by using different versions of the digit span task. In 12 studies (Danahy et al., 2007; Bialystok and Feng, 2009; Bialystok and Viswanathan, 2009; Engel de Abreu, 2011; Kapa and Colombo, 2013; Engel de Abreu et al., 2014; Filippi et al., 2015; Garraffa et al., 2015; Cockcroft, 2016; Blom et al., 2017; Raudszus et al., 2018; Veenstra et al., 2018) no significant differences between the two groups emerged. In three studies, monolinguals remembered a significantly higher number of digits than bilinguals in the forward digit span task (Buac et al., 2016; Bosman and Janssen, 2017) and backward digit span (Jaekel et al., 2019). In Bialystok (2010), which reported three studies involving three different groups of participants, bilinguals' scores were lower than monolinguals' scores only in the third study. In this study, bilingual participants had a smaller vocabulary size when compared to monolinguals. In Blom et al. (2014), bilinguals scored were higher in both forward and backward digit span. In Hartanto et al. (2019), which assessed the performance in four different time waves, bilinguals had better performance than monolinguals only in time 4 (mean age bilinguals: 7.13; mean age monolinguals: 7.05).

Three studies (Engel de Abreu, 2011; Garraffa et al., 2015; Cockcroft, 2016) evaluated short-term verbal memory using the non-word repetition task. In Engel de Abreu (2011), the monolinguals repeated a significantly higher number of non-word than bilinguals. To verify whether the difference in vocabulary size between participants affected the results, the author repeated the analysis using the receptive vocabulary score as a covariate, and the difference between the two groups disappeared. In the other two studies, there were no significant differences in the performance of the two groups.

Arizmendi et al. (2018) used the number updating task, and no differences emerged between the two groups of participants.

#### Bilingualism and Inhibition (n = 28)

Two studies (Bonifacci et al., 2011; Barac et al., 2016) used the Go/No-Go Task. In Barac et al. (2016), bilinguals were faster and more accurate than monolinguals. The d' index indicated a better discriminatory capacity in the bilingual group. In Bonifacci et al. (2011), which used a modified version of the Go/No-Go task, the No-Go condition consisted of an image accompanied by a sound; the two groups were equal on the number of omissions, the percentage of accuracy and the RTs.

Two studies (Arizmendi et al., 2018; Dick et al., 2019) used the Stop-Signal task, and no differences between the performances of the two groups emerged.

Nine studies (Gathercole et al., 2010; Duñabeitia et al., 2014; Mohades et al., 2014; Abdelgafar and Moawad, 2015; Jalali-Moghadam and Kormi-Nouri, 2015; Schröter and Schroeder, 2017; Arizmendi et al., 2018; Escobar et al., 2018; Nayak et al., 2020) assessed cognitive inhibition by using the Stroop task (Stroop, 1935). Two studies (Abdelgafar and Moawad, 2015; Jalali-Moghadam and Kormi-Nouri, 2015) used the pencil and paper version of this task and did not find any significant difference in the performance of monolingual or bilingual participants. Two studies (Duñabeitia et al., 2014; Schröter and Schroeder, 2017) adopted the computerized version of the task, and no significant differences between the groups occurred. In two studies (Duñabeitia et al., 2014; Mohades et al., 2014), a modified version of the task with numerical stimuli was adopted. In this task, children had to report which number was larger, ignoring the physical size of the digits. In Duñabeitia et al. (2014), no significant differences between the groups were found. In Mohades et al. (2014), no significant differences between the groups were found for RTs and accuracy, but the bilingual group showed a higher congruency effect. Nayak et al. (2020) used animal stimuli and did not find significant differences between the two groups, even after controlling for age and socioeconomic status. In Gathercole et al. (2010), monolingual participants solved the classic Stroop task in English while bilinguals carried out the task in both English and Welsh. There were no significant differences among the three groups of bilinguals in both accuracy and reaction times in the Welsh version. Significant differences in accuracy score in the primary school age group emerged in the English version. The comparison among the three bilingual groups showed a lower accuracy in the group exposed at home to Welsh for 80% of the time from birth (OWH). Monolinguals had significantly fewer accuracy scores than those exposed to both Welsh and English at home from birth (WEH). For reaction times, significant differences emerged only in the teens, and monolingual participants responded significantly slower than all bilingual groups. Escobar et al. (2018) used the Day-Night Stroop Task. The experimental task included congruent trials in which participants named the word corresponding to the presented stimulus (e.g., the word day for the sun) and incongruent trials in which they had to pronounce the word opposite to the presented stimulus (e.g., the word day for the moon). No significant differences emerged between the two groups. In Arizmendi et al. (2018), two modified versions of the Stroop task were used. In both versions, participants had to respond orally. No significant differences emerged between bilinguals and monolinguals.

Nine studies (Engel de Abreu et al., 2012, 2014; Calvo and Bialystok, 2014; Poarch and Bialystok, 2015; Blom et al., 2017; Ross and Melinger, 2017; Park et al., 2018; Struys et al., 2018; Dick et al., 2019) evaluated the interference suppression ability using the Flanker task (Eriksen and Eriksen, 1974). In four studies (Engel de Abreu et al., 2012, 2014; Poarch and Bialystok, 2015; Park et al., 2018), bilingual participants had faster RTs. In two studies (Poarch and Bialystok, 2015; Park et al., 2018), this advantage emerged in the incongruent condition indicating a better ability to control conflictual information in the bilingual group.

In Blom et al. (2017), the performance in the Flanker task correlated negatively with the scores in memory tasks, indicating that children with better results in memory tasks had faster reaction times. Moreover, multiple linear regression results have suggested that a more extended vocabulary size is associated with a better ability to perform this experimental task. However, no significant differences between monolinguals and bilinguals emerged. Three studies (Calvo and Bialystok, 2014; Ross and Melinger, 2017; Dick et al., 2019) showed no significant difference in RTs between bilinguals and monolinguals, but in Calvo and Bialystok (2014) bilinguals reached a higher percentage of accuracy. Struys et al. (2018) analyzed the speed-accuracy trade-off effect (i.e., an increase in accuracy corresponds to an increase in reaction times and vice versa) to verify whether the participants adopted different resolution strategies in the experimental tasks. The results indicated a speed-accuracy trade-off effect in the older bilingual group (mean age: 11.7) but not in the younger bilingual group (mean age: 6.6) or in the monolingual groups. The authors hypothesized that the effect was not present in both groups of bilinguals because they may have adopted different strategies (preferring speed in some cases and accuracy in others). To highlight an advantage in the speed-accuracy trade-off effect, it seems necessary that most participants adopt the same strategy.

Seven studies analyzed the ability to manage conflictual information by using the flanker task in the experimental context of the Attentional Network Test (Carlson and Meltzoff, 2008; Kapa and Colombo, 2013; Antón et al., 2014; Ladas et al., 2015; Barac et al., 2016; Yang and Yang, 2016; Veenstra et al., 2018). In two studies (Antón et al., 2014; Ladas et al., 2015), no significant differences in reaction times and the percentage of accuracy between the monolingual and bilingual groups were observed. In the other two studies (Barac et al., 2016; Yang and Yang, 2016), no significant differences in reaction times emerged, while bilinguals were more accurate in congruent and incongruent trials than the monolingual group. In three studies (Carlson and Meltzoff, 2008; Kapa and Colombo, 2013; Veenstra et al., 2018), the Flanker x Group interaction results were not reported.

Seven studies (Poarch and van Hell, 2012; Gathercole et al., 2014; Mohades et al., 2014; Ross and Melinger, 2017; Raudszus et al., 2018; Struys et al., 2018; Zeng et al., 2019) used the Simon Task (Simon and Wolf, 1963). In two studies (Poarch and van Hell, 2012; Raudszus et al., 2018), no significant differences emerged between the monolingual and the bilingual groups. Two studies (Ross and Melinger, 2017; Zeng et al., 2019) found a lower percentage of errors in the bilingual group than to the monolingual group, while there were no differences between the two groups in reaction times and the Simon effect. In Gathercole et al. (2014), there were no significant differences between monolinguals and bilinguals in the primary schoolers and teens groups. In the group of 5-year-olds, no difference emerged for the percentage of accuracy. However, the monolinguals were faster than the bilingual group exposed at home to English for 80% of the time from birth (OEH). The OWH bilinguals were faster than the OEH bilinguals. In Mohades et al. (2014), bilinguals achieved the same performance as monolinguals in reaction times and accuracy, but they showed a greater congruency effect. In Struys et al. (2018), a speed-accuracy trade-off effect occurred in the two groups of bilinguals but not in monolingual participants.

Three studies (Bialystok, 2010; Cottini et al., 2015; Arizmendi et al., 2018) assessed inhibition using the Global Local Task (Andres and Fernandes, 2006). In Bialystok (2010), the Global-Local task was proposed in three different versions. Overall, bilinguals were faster under all conditions than monolinguals. Bilinguals were more accurate than monolinguals in the global condition while in the local condition, there was no difference between the two groups. Moreover, the mixing costs (the difference between trials alone and trials in mixed condition) were smaller for bilinguals than for monolinguals. In Cottini et al. (2015), bilinguals were more accurate than monolinguals in incongruent and neutral trials, and the total effect of interference was higher in the monolingual group. In this study, bilinguals were more accurate than monolinguals in the local incongruent trials, while monolinguals performed significantly better than bilinguals in the global incongruent trials. In Arizmendi et al. (2018), no significant differences were found between monolingual and bilingual participants.

Two studies (Carlson and Meltzoff, 2008; Barac et al., 2016) used a delay gratification task to assess the ability to inhibit dominant responses. In both studies, no significant differences were found between the monolingual and bilingual participants.

Two studies (Garraffa et al., 2015; Schröter and Schroeder, 2017) used the Opposite World Task from the Test of Everyday Attention for Children (Manly et al., 2001) in which it is required to inhibit a dominant verbal response. In Garraffa et al. (2015), bilinguals were slower than monolinguals, while in Schröter and Schroeder (2017), no significant difference between the two groups emerged.

#### Bilingualism and Shifting (n = 12)

Two studies (Barac and Bialystok, 2012; Veenstra et al., 2018) used the Color-Shape task switching. In Barac and Bialystok (2012), bilinguals were faster and had lower global costs than monolinguals. In Veenstra et al. (2018), which used a composite inhibition score, considering the ANT interference effect, no significant differences emerged between bilinguals and monolinguals. Arizmendi et al. (2018) used a modified version of the Color-Shape task, the Pirate Sorting task, and did not find significant differences between the two groups.

Six studies (Bialystok, 1999; Carlson and Meltzoff, 2008; Garraffa et al., 2015; Escobar et al., 2018; Park et al., 2018; Hartanto et al., 2019) used different versions of the Dimensional Change Card Sort Task (e.g., Zelazo et al., 1996). In four studies (Bialystok, 1999; Carlson and Meltzoff, 2008; Garraffa et al., 2015; Hartanto et al., 2019), the bilingual group gave more correct responses than the monolingual group. In Park et al. (2018), bilinguals showed lower mixing costs (the difference between trials in the pre-shift condition and non-switch trials in the mixed condition) compared to monolinguals, while no significant difference emerged between the two groups in the switching costs (the difference between non-switch and switch trials in the mixed condition) and shifting costs (the difference between the pre-shift and the post-shift condition). Escobar et al. (2018) found no differences between the two groups.

Gathercole et al. (2014) used a modified card task. In the teen group, the OWH bilingual group was more accurate than the monolinguals and WEH bilinguals. Monolinguals were faster in the group of 5 years old, whereas bilinguals were faster in the group of teenagers.

Ross and Melinger (2017) used a modified version of the Wisconsin Card Sorting Test, the Berg Card Sorting Test (Piper et al., 2012) and did not find differences between the two groups in perseverative errors, reaction times or the number of trials needed to complete a category. However, bilinguals made more total errors than monolinguals.

Gathercole et al. (2010) used the Tapping Task. Three groups of bilinguals who used different languages at home were included in the study. In the primary age group, the OWH and OEH groups showed better performance in the match condition (i.e., emulation of the experimenter's action) and the switch condition (i.e., to do actions contrary to those of the experimenter). In the teen group, the OWH and WEH groups showed an advantage over the monolingual group.

#### Bilingualism and Multiple Executive Functions (n = 10)

This section examines the results of experimental tasks that evaluated different executive functions at the same time.

Three studies (Bialystok and Viswanathan, 2009; Bialystok, 2010; Abdelgafar and Moawad, 2015) used the Trail Making Test, a neuropsychological test that allows evaluating visual attention and switching ability. In all studies, bilinguals completed part A faster than monolinguals. In two studies (Bialystok and Viswanathan, 2009; Bialystok, 2010), bilinguals solved part B faster.

Five studies (Bialystok, 2010; Abdelgafar and Moawad, 2015; Friesen et al., 2015; Escobar et al., 2018; Zeng et al., 2019) used the verbal fluency task. Verbal fluencies require linguistic ability and executive control during lexical access. In the semantic version of this task, the number of possible responses is higher, requiring a high degree of executive control. This result is due to the need to inhibit spontaneous associations not inherent to the criterion and to comply with the restrictions such as the morphological ones (Friesen et al., 2015). In Abdelgafar and Moawad (2015), semantic fluency was considered an indicator of inhibition ability while in Bialystok (2010), categorical fluency was considered a verbal productivity indicator. In both studies, no significant differences between the two groups emerged. Conversely, in the other two studies (Escobar et al., 2018; Zeng et al., 2019), bilinguals produced more words than monolinguals in letter fluency tasks. In Escobar et al. (2018), bilinguals produced more words even in the semantic fluency task. In Friesen et al. (2015), the authors argue that for the performance of the task, it is necessary to involve different components of the executive functions. In terms of categorical fluency, 10-year-old bilingual children produced fewer words than monolinguals. There was no difference in semantic fluency. For the 7-year-old group, there was no difference in both types of verbal fluency between the two groups. However, bilingual children had a higher mean subsequent-response latency, that is, the time in which half of the responses were produced. This index could indicate a difficulty for bilinguals in the lexical access due to the interference produced by the two languages known.

Bialystok and Viswanathan (2009) used the Face Task (Bialystok et al., 2006) to evaluate simultaneously three components of executive functions, i.e., response suppression, inhibitory control, and cognitive flexibility. No significant differences in the performance of the three groups (two bilingual and one monolingual groups) were found considering both response suppression and accuracy. Monolinguals had higher inhibitory and switching costs than bilinguals. The two bilingual groups evaluated in this study did not differ.

Bialystok (2011) used the Dual modality classification task, an experimental task in which stimuli can be visual and auditory. In the single-modality condition, no significant differences in the performance of the two groups emerged. In the dual-modality condition, bilinguals had a higher accuracy score.

Krizman et al. (2016) used the Integrated Visual and Auditory Continuous Performance Test. Participants were required to respond or inhibit the response depending on the specific auditory or visual stimulus presented. Bilinguals performed better than monolinguals. Furthermore, low-SES bilinguals performed better than low-SES monolinguals and at the same level as participants with high SES.

Carlson and Meltzoff (2008) used a modified version of the Kansas Reflectory/Impulsivity Scale (KRISP; Wright, 1971), Statue (Korkman et al., 1998), Simon says (Strommen, 1973), and the Gift Delay. These tasks require to suppress motor action during a delay. No significant differences emerged between the bilingual and monolingual groups.

Jaekel et al. (2019) used the Hearts and Flowers task. No significant differences emerged between the bilingual and monolingual groups.

## Discussion

Bilingualism is the knowledge of two languages. Given the absence of a single definition, it is possible to consider bilinguals with a different degrees of proficiency in the languages they know or who have learned languages in different contexts, such as school or home, or different periods of their lives. According to the Joint Activation Model of Green (1998), bilingualism involves the activation of both languages in the brain, even when only one language is used. This condition seems to have a positive effect on several cognitive functions, including executive functions (Bialystok et al., 2012). After the publication of positive evidence on the bilingual effect, this hypothesis was questioned, given the difficulty in replicating the previous results. This difficulty seems to be due to particular circumstances in which different factors (e.g., age of participants, socioeconomic status, experimental tasks) are involved (i.e., Paap et al., 2015).

The current systematic review summarizes the results of 53 studies published between 1999 and 2020 that investigated the effect of bilingualism on executive functions. Analyzing the selected studies, it emerged that the participants had very different characteristics and wide variability in the sample size, ranging from a minimum of 12 participants (Carlson and Meltzoff, 2008) to a maximum of 1740 (Dick et al., 2019). Furthermore, the studies adopted various tasks for the assessment of executive functions. These methodological differences could explain the mixed results found, making it difficult to draw definitive conclusions about the existence of the bilingual effect.

Evidence supporting the existence of the bilingual effect appears when inhibitory control and cognitive flexibility are assessed. In particular, the Sky Search task, the Flanker task, the Dimensional Change Card Sort task, and the Trail Making Test seem to indicate the existence of a bilingual effect. A deeper analysis of the characteristics of the studies included reveals several differences that should lead to a cautious interpretation of the results. The great variability of the experimental tasks becomes evident when considering the studies that used the Stroop task. In particular, the nine studies adopted six different versions of the task. Six studies used different versions of the task with verbal stimuli (i.e., pencil-paper version; computerized version; oral responses version), and found no significant differences between different groups. Two studies used two different versions with non-verbal stimuli, and no significant differences emerged between monolinguals and bilinguals. Two studies used the numerical version, and mixed results were found. However, determining the degree of incidence of the type of stimulus is not possible since no study included both verbal and non-verbal versions of the task. Furthermore, it is not possible to exclude the incidence of the linguistic aspect in the numerical version of the task. As pointed out by Duñabeitia et al. (2014), it is possible that the linguistic representations of the numbers in the two known languages were active in bilingual brains, and the same may have happened in the non-verbal version since stimuli were used that can be easily verbalized.

Different versions of the task were included in the studies that adopted the Flanker task. The most variable feature was the type of stimulus used (i.e., fish; chevron). Mixed results also emerged in three studies where the same version of the Flanker task was used. Two studies (Engel de Abreu et al., 2012, 2014) confirmed the bilingual effect, while in Blom et al. (2017) no significant differences emerged. It can be hypothesized that the mixed results may be caused by differences in the participants' linguistic and cultural backgrounds. In two studies (Engel de Abreu et al., 2012, 2014) bilingual participants were recruited in the Grand Duchy of Luxembourg, a trilingual country with a trilingual education system where children start formal education in the first language at age 4, are exposed to the second language at age six and to the third language at age 7. As the participants in the studies were, on average, eight years old, the bilingual participants included participants that could be considered “trilingual.” In Blom et al. (2017), three groups of bilingual participants who knew three different language pairs were included. The monolinguals' characteristics may also have influenced the results since, in two studies (Engel de Abreu et al., 2012, 2014), they were recruited in a different country than the bilinguals. It cannot be excluded that cultural aspects influenced the results.

Most studies that used ANT to evaluate attentive networks did not reveal significant differences between the monolingual and bilingual groups. Again, different factors may have influenced the results. Some authors (e.g., Mullane et al., 2016; Lewis et al., 2018) highlighted that the child version of the ANT could generate a lower interference effect than the adult version despite the fact that increasing the level of motivation of children to perform the experimental task. When children are evaluated with the adult version, developmental differences emerge that are not visible with the child version. Future studies may adopt the adult version for the assessment of attention in bilinguals. In Yang and Yang (2016), which found faster reaction times and better accuracy in bilinguals, bilingual participants' cultural. and linguistic background may have influenced the results. Bilingual participants knew a language pair composed of two languages belonging to two different language families, characterized by significant orthographic differences (i.e., Korean-English). This factor seems to have a positive effect on visuospatial abilities (Yang and Yang, 2016). Furthermore, belonging to certain cultures (e.g., Chinese culture) seems to positively influence the development of executive functions (Carlson and Meltzoff, 2008). Also, in Kapa and Colombo (2013), the bilingual participants' characteristics seem to have a role in the differences that emerged. In the study, the early bilinguals showed better attentive abilities than the monolinguals, but this advantage did not characterize the late bilinguals.

Even in the studies that evaluated the shifting ability with DCCS, some conflicting results emerged. In Park et al. (2018), significant differences in reaction times emerged between the two groups of participants in the most demanding condition. Other studies using this task confirmed a bilingualism effect. However, it is important to note that in almost all the other studies only the participants' accuracy was assessed. The study of Park et al. (2018) would indicate that the task is too simple for the age considered: the participants included in this study were older compared to the other studies. In Escobar et al. (2018), the bilinguals had faster reaction times than the monolinguals, but this difference was not significant. The small number of participants (i.e., 17 bilinguals and 17 monolinguals) may have reduced the statistical power of the results.

Another task that showed mixed results is the verbal fluency task. Once again, it is important to highlight that the studies included adopted different versions of this task. Most of the studies that assessed executive functions using category fluency required the participants to name words belonging to the “animals” category. Friesen et al. (2015) used the category “clothing items.” This factor seems to have influenced the results since only in Friesen et al. (2015) did the monolingual group outperformed the bilinguals, whereas, in the other studies, there were no significant differences between the two groups or better performance in the bilinguals. Regarding the letter fluency, several methodological differences emerged. The studies adopted different letters, modalities of administration of the task (oral vs. written production), duration of the test (5 min vs. 1 min), or modalities of calculation of the final score (inclusion or exclusion of incorrect words). Concerning verbal and visual working memory, the evidence for better performance of the bilingual group is limited. In some studies, bilingual participants presented lower performance than monolinguals in the verbal working memory. This result would seem to be mediated by the linguistic abilities of the participants: in Bialystok (2010), bilinguals showed worse performance than monolinguals only when bilinguals showed a reduced vocabulary size than monolinguals.

Ladas et al. (2015) suggested that, in experimental tasks using verbal stimuli, the absence of a significant result could be interpreted as a bilingual advantage because it is well-known that the vocabulary size of bilinguals, if it is calculated considering only one language, is reduced when compared to that of monolinguals. For example, in Blom et al. (2014), when the difference in vocabulary size was statistically controlled, a bilingual effect emerged in both the Dot Matrix task and the Digit Backward Recall. However, the absence of significant differences in the performance of bilinguals and monolinguals also emerges in non-verbal tasks, and sometimes even studies using the same experimental task did not observe the same results. These findings suggest that other factors, such as the characteristics of the experimental tasks and the participants, influence the results. The wide variety of tests used for assessing executive functions, which are frequently modified by research groups, makes it difficult to compare the results directly. In several cases, a specific test is used in a single study, or when more than one experimental task is used, the tests chosen had low convergent validity. As suggested by Paap et al. (2015), each study should include a minimum of two tasks to evaluate each executive function. This methodological choice would make it possible to confirm that controlling that the results are not due to task-specific characteristics. Another point to clarify is whether the bilingual effect only emerges when the task requires a specific degree of complexity or the coordination of several executive functions. In Barac et al. (2016), which included tasks of increasing difficulty, no differences were observed in the easier task (gift delay), while bilinguals showed an advantage in the more complex tasks (Flanker task and Go/No-Go task). Conversely, in the studies using the Corsi test, the bilingual effect emerges only when an easier version of the task was used (Frog Task Matrix).

The studies included in this systematic review provide an overview of the variability of the population considered in studies on bilingualism. Some studies include bilingual participants who know different language pairs (e.g., Engel de Abreu, 2011; Friesen et al., 2015), and other participants who are children of immigrants who may face different cultural, family and social contexts (e.g., Leikin and Tovli, 2014; Ladas et al., 2015). Moreover, information about the acquisition and the use of known languages is not always given, and it does not allow determining the type of bilingualism (i.e., simultaneous or sequential) or the interactional context. Information, such as the age of acquisition of the first and second language, the degree of exposure, and the daily use of the languages, would lead to select better bilinguals. It could allow verifying the possible effects of these characteristics. Knowing the same languages does not determine having shared the same bilingual experience because the interactional contexts in which languages are used may not be the same (Antoniou, 2019). Most studies included in this review do not include information about the context in which language exchanges occur, and linguistic contexts can be very different.

For the classification of participants in bilinguals and monolinguals, parental and self-reports are usually used as they are considered reliable instruments for evaluating experience related to second language acquisition (Gutiérrez–Clellen and Kreiter, 2003; Bedore et al., 2011). The lack of detailed information about the bilingual experience could lead to an incorrect classification of the participants, not allowing them to detect any differences. This problem is highlighted by Poarch and Bialystok (2015), who included a group of partial bilinguals (i.e., native speakers of English who had been learning French for about 2 years) that achieved the same performance as monolinguals. The inclusion of these participants in the bilingual group would have nullified the difference in performance between bilinguals and monolinguals. Another aspect to consider is when children begin formal school education. When children begin school, they are exposed to one or more foreign languages depending on the educational program. Therefore, information on the weekly frequency of exposure and use of the foreign language should be collected.

Some sociodemographic factors, such as low socioeconomic or immigrant status, affect the development of executive functions. Frequently migrant population has a low socioeconomic status, and their bilingualism is often secondary to the migration in a foreign country. In America, there is a high association between low SES and bilingualism. Several studies confirm that belonging to families with low socioeconomic status has negative consequences on the development of different cognitive functions and language skills. In this adverse situation, bilingualism seems to act as a protective factor (Hartanto et al., 2019); in fact, some studies (e.g., Engel de Abreu et al., 2012; Krizman et al., 2016) reported an advantage of bilingual participants when the socioeconomic status was controlled. The cognitive advantage of bilingualism can be developed independently by the SES (Blom et al., 2014; Calvo and Bialystok, 2014).

Further, it needs to clarify at which specific point in the lifespan the bilingual effect should be studied. The strongest evidence supporting the bilingual effect comes from studies that have included participants with executive functions that are not at a maximum level (e.g., older people). The bilingual effect should be evident in children because they have not yet reached the full development of cognitive functions (Antón et al., 2014). Most of the studies in this review investigated the existence of the bilingual effect in children between 5 and 9 years of age. Only thirteen studies included early adolescent participants (10–14 years), while none included middle adolescent participants (15–17 years). The longitudinal study by Park et al. (2018) showed that results could be influenced by time points when individuals are tested and that the various components of the executive functions would seem to follow different trajectories of development. In this study, the bilinguals and monolinguals achieved the same performance when individuals were tested for updating abilities while a bilingual effect in inhibition skills emerged at time 2 but not at time 1. Finally, an advantage was found for the bilingual group in terms of shifting abilities at both times 1 and 2 for mixing cost, while no advantage was found for shifting and switching cost. In addition to age, the test used would also seem to influence the results: in Struys et al. (2018) in which groups of participants of different ages were compared, a smaller congruency effect was found in the group of younger bilinguals (mean age 6.6 years) on the Simon task and a smaller congruency effect for older bilinguals (mean age 11.7 years) on the flanker task. Longitudinal studies should be conducted to investigate whether bilingualism affects the development trajectories of executive functions. It is still unclear how much “training” of the executive functions (in terms of years or time spent on the use of the two languages) is necessary to produce a difference between bilinguals and monolinguals and, therefore, when the condition of bilingualism generates an advantage.

## Limitations

This systematic review of the literature has not reached a definitive conclusion about the bilingual effect. This limitation is due to the high variability of the results observed by the different studies. Moreover, as Leivada et al. (2020) recently pointed out, systematic reviews assume that a comparison is made among studies that include similar populations, which is often not the case with these bilingual studies. In the studies on bilingualism, the adoption of a dichotomous “monolingual vs. bilingual” approach and the absence of a shared definition of bilingualism has led to an oversimplification of reality and the inclusion of individuals with very different characteristics in the same group. DeLuca et al. (2019) suggested the need to consider bilingualism as a spectrum of experiences that can affect neural plasticity. Moreover, the monolingual group also presents a degree of variability that should not be ignored (Baum and Titone, 2014). Several aspects of the experience of individuals or groups would seem to affect brain adaptation differently. A quantitative analysis of the literature would have allowed stronger conclusions, but it was impossible to use a metanalytic approach because of the variability of the experimental tasks adopted in the different researches. Comparing the effects size and statistical analysis of the various studies could help to understand the results better. Future studies should analyze the characteristics of the participants more, and verify which factors, such as the AoA or the daily use of each language, influence the results.

## Conclusions

The results summarized in this systematic review indicate the need for further studies that should consider the factors that have been identified as possible modulators of the observed results. Future studies should provide more information about the language context in which bilingual participants are immersed. It would be useful to establish guidelines identifying the minimum information needed to be included in the studies for the description of the bilingual population. Several researchers have highlighted the need to adopt a new approach to the study of this topic. Large-scale research projects involving several laboratories worldwide would provide clearer answers about the existence of a positive effect of bilingualism and identify the variables involved in this process (Baum and Titone, 2014; Leivada et al., 2020). From the summary of the studies included in this systematic review, it emerges that current evidence does not make it possible to establish the existence of a bilingual effect or to identify the factors involved in determining the bilingual effect. Since bilingualism is a reality concerning a substantial percentage of the population, it is important to clarify this topic. A result in favor of the existence of the bilingual effect would provide the incentive for the implementation of bilingual school programs that could lead to extensive and regular use of more than one language. On the contrary, a reduction in performance linked to the condition of bilingualism would indicate the need to develop support programs aimed at those who, due to various circumstances, such as immigrant status or bilingual school education, are facing this situation. Executive functions are included in life skills, i.e., psychosocial skills that, if properly trained, enable the prevention of social and health problems, the promotion of social and personal development, and the protection of human rights. The absence of specific tests for the evaluation of bilinguals suggests the need to develop *ad hoc* instruments or to provide the validation of existing tests for this specific population. Tests containing verbal stimuli, used to make diagnoses, could lead to an overestimation of the problems. It would be useful to conduct a further systematic review focusing on the adult population to analyze the effect of bilingualism on those who have reached a peak or are in a phase of decline of executive functions.

## Data Availability Statement

The original contributions presented in the study are included in the article/supplementary material, further inquiries can be directed to the corresponding author/s.

## Author Contributions

JG and MC: conceptualization of the review, literature search, writing of the original draft, revision, and editing of the manuscript. DM: conceptualization of the review, writing of the original draft, revision, and editing. FF and SP: revision and editing of the manuscript. All authors contributed to the article and approved the submitted version.

## Conflict of Interest

The authors declare that the research was conducted in the absence of any commercial or financial relationships that could be construed as a potential conflict of interest.
